# Frequent detection of monkeypox virus DNA in saliva, semen, and other clinical samples from 12 patients, Barcelona, Spain, May to June 2022

**DOI:** 10.2807/1560-7917.ES.2022.27.28.2200503

**Published:** 2022-07-14

**Authors:** Aida Peiró-Mestres, Irene Fuertes, Daniel Camprubí-Ferrer, María Ángeles Marcos, Anna Vilella, Mireia Navarro, Laura Rodriguez-Elena, Josep Riera, Alba Català, Miguel J Martínez, Jose L Blanco, on behalf of the Hospital Clinic de Barcelona Monkeypox Study Group

**Affiliations:** 1 Barcelona Institute for Global Health (ISGlobal), Hospital Clínic-Universitat de Barcelona, Barcelona, Spain; 2 Dermatology Department, Hospital Clínic-Universitat de Barcelona, Barcelona, Spain; 3 Microbiology Department, Hospital Clínic-Universitat de Barcelona, Barcelona, Spain; 4 Centro de Investigación Biomédica en Red de Enfermedades Infecciosas (CIBERINFEC), Instituto de Salud Carlos III, Madrid, Spain; 5 Preventive Medicine and Epidemiology Department, Hospital Clínic-Universitat de Barcelona, Barcelona, Spain; 6 Infectious Diseases Department, Hospital Clínic-IDIBAPS, Universitat de Barcelona, Barcelona, Spain; 7 Additional members of the Hospital Clinic de Barcelona Monkeypox Study Group are listed under Acknowledgements

**Keywords:** monkeypox, shedding, saliva, semen, outbreak, PCR

## Abstract

A monkeypox (MPX) outbreak has expanded worldwide since May 2022. We tested 147 clinical samples collected at different time points from 12 patients by real-time PCR. MPX DNA was detected in saliva from all cases, sometimes with high viral loads. Other samples were frequently positive: rectal swab (11/12 cases), nasopharyngeal swab (10/12 cases), semen (7/9 cases), urine (9/12 cases) and faeces (8/12 cases). These results improve knowledge on virus shedding and the possible role of bodily fluids in disease transmission.

Up to 11 July 2022, more than 9,000 cases of monkeypox (MPX) have been confirmed in 57 countries [1]. The analysis of seven cases diagnosed in the United Kingdom between 2018 and 2021 revealed prolonged detection of MPX virus DNA in nasopharyngeal swabs, urine and blood samples [2]. In addition, a report of four cases in Italy from the current outbreak detected MPX DNA in semen, faeces and saliva [3]. We aimed to characterise viral shedding to better understand the possible role of different bodily fluids in disease transmission and investigated the presence of MPX virus DNA in saliva, rectal swab, nasopharyngeal swab, semen, urine and faecal samples, from 12 MPX patients in Barcelona, Spain.

## Sample collection

During May and June 2022, patients presenting with a clinical suspicion of MPX infection at a tertiary hospital in Barcelona were examined in an isolation room, where samples from two different lesions were collected for diagnosis. In parallel, samples were taken for the screening of other STIs: *Neisseria gonorrhoeae*/*Chlamydia trachomatis*/*Mycoplasma genitalium* in pharynx, urine and rectum by PCR; *Treponema pallidum*/*Lymphogranuloma venereum*/herpes in genital-anal ulcers by PCR and hepatitis B virus (HBV)/hepatitis C virus (HCV)/HIV/syphilis by serology. Upon MPX laboratory confirmation, 17 patients were invited to participate in our study, which included collection of saliva, rectal swab, nasopharyngeal swab, semen, urine and faecal samples; 12 accepted and 5 declined participation. When possible, more than one time point of follow-up samples per patient was obtained. Clinical and epidemiological data were retrieved from the medical records and retrospectively reviewed. 

## Patient characteristics

All patients studied (n = 12) were young adult men who have sex with men (MSM; median age: 38.5 years; range: 32–52). Most (9/12) had previous history of sexually transmitted infections (STIs), and three patients had a concomitant STI. Four were HIV-positive (all with undetectable HIV viral load and CD4^+^ T-cell counts between 400 and 860 cells/uL). All patients were sexually active with up to 10 sexual partners during the last month and seven were on HIV pre-exposure prophylaxis. Five patients reported attendance at ‘sex-on-premises’ venues or ‘chemsex’ sessions. Three reported trips to other parts of Spain but none reported a visit to the Canary Islands or Madrid, where transmission of MPX was traced initially in Spain. Four patients reported previous sexual contact with a confirmed case of MPX. An unspecific systemic syndrome (fever, myalgia, general malaise, etc) was reported by 11 of 12 patients. In half of the patients, the skin lesions were present in more than one location of the body. Four reported smallpox vaccination, whereas this vaccine information was not recorded in five. The clinical and epidemiological characteristics of the patients included are shown in Table 1. 

**Table 1:** Clinical and epidemiological characteristics of studied monkeypox patients^a^, Barcelona, Spain, May–June 2022 (n = 12)

Characteristics	Pat. 01	Pat. 02	Pat. 03	Pat. 04	Pat. 05	Pat. 06	Pat. 07	Pat. 08	Pat. 09	Pat. 10	Pat. 11	Pat. 12
Age (years)	30s	30s	40s	40s	40s	30s	40s	50s	40s	30s	30s	30s
Concomitant STIs	No	Syphilis	No	No	No	No	No	Syphilis	No	No	No	CT, NG
HIV status	Neg	Pos	Pos	Neg, on PrEP	Neg, on PrEP	Neg, on PrEP	Neg, on PrEP	Pos	Pos	Neg, on PrEP	Neg, on PrEP	Neg, on PrEP
Number of sexual partners (last month)	5–10	5–10	5–10	5–10	5–10	< 5	< 5	5–10	< 5	< 5	5–10	5–10
Epidemiological link^a^ (Travel/contact)	No / Yes	No / Yes	Spain / No	Spain / No	Italy / No	NA / No	No / No	Spain / No	No / Yes	No / No	No / Yes	No / No
Localisation of skin lesions	Arm, perianal area and trunk	Genital area	Anal area	Perianal, chest and trunk	Chest and legs	Wrist, pectoral, fingers, hand and perianal area	Ulcerated ventral tongue	Trunk and genital area	Genital lesions	Perianal area	Genital area	Genital and anal area
Other symptoms	Myalgia, fatigue	Odynophagia, GM	Myalgia, fever, Proctitis	Proctalgia, odynophagia, GM	Fever, myalgia, GM	Fever, proctitis	Headache, GM	GM, fever	Myalgia, GM	GM, myalgia, proctitis	NA	Myalgia, GM
Smallpox vaccine	NA	NA	NA	NA	Yes	NA	No	Yes	Yes	No	Yes	No

CT: *C. trachomatis;* GM: general malaise; NA: not available; Neg: negative; NG: *Neisseria gonorrhoeae*; Pat.: patient; Pos: positive; PreP: pre-exposure prophylaxis; STI: sexually transmitted infection.

^a^ All patients were men who have sex with men.

^b^ Epidemiological links were investigated. Travel: History of travel outside Barcelona in the last month/ contact: history of close physical contact with a MPX positive case.

## Laboratory methods for monkeypox virus detection

Clinical samples (saliva, rectal and nasopharyngeal swab, semen, urine and faecal samples) were inactivated with a 1:1 volume of cobas omni lysis reagent (Roche Diagnostics, Mannheim, Germany) prior to nucleic acid extraction in an automated system (MagNA Pure Compact, Roche Diagnostics) using the MagNA Pure Compact Nucleic Acid Isolation Kit I—Large Volume. Stool samples were processed as previously described [4]. Upon the initial MPX diagnosis, all samples tested positive with a commercial orthopox generic real-time PCR assay (Lightmix Roche Diagnostics, Mannheim, Germany) and with a specific MPX real-time PCR protocol [5]. In addition, the first three diagnosed MPX cases were confirmed by Sanger sequencing of a PCR amplicon, amplified as previously described [6]. All follow-up samples were tested with the specific MPX PCR assay.

## Viral shedding in clinical samples 

At the time of diagnosis, MPX DNA was detected in swabs of skin lesions in all 12 cases (Table 2). In most cases (9/12), high viral loads (quantification cycle (Cq) value range: 16–21) were observed in skin pustules and some patients presented with additional oral, pharyngeal and rectal lesions.

**Table 2:** Clinical sample characteristics of 12 monkeypox patients at diagnosis by collection day since symptom onset and qPCR results, Barcelona, Spain, May–June 2022 (n = 18 samples)

Patient	Days since symptom onset	Skin lesions n = 12	Other samples n = 6
Pat. 01	6	Pos (21.3)	NA
Pat. 02	5	Pos (18.5)	NA
Pat. 03	1	Pos (16.2)	NA
Pat. 04	7	Pos (17.6)	RS: Pos (17.8)
Pat. 05	14	Pos (23.2)	NA
Pat. 06	7	Pos (22.1)	RS: Pos (16.1)
Pat. 07	4	Pos (21.3)	TU: Pos (19.6)
Pat. 08	3	Pos (27.8)	OL: Pos (35.8)
Pat. 09	7	Pos (19.0)	NA
Pat. 10	2	Pos (19.0)	RS: Pos (17.7)
Pat. 11	1	Pos (18.8)	NA
Pat. 12	3	Pos (21.1)	RS: Pos (15.6)

NA: not applicable; Neg: monkeypox DNA not detected; Pat.: monkeypox patient; Pos: positive, monkeypox DNA detected; RS: rectal swab; TU: tongue ulcer; OL: oral lesion.

Quantification cycle (Cq) values are indicated in brackets after positive results.

In all cases, MPX DNA was detected in several follow-up samples taken between 4 and 16 days post-symptom onset (Table 3), and in one third (Patients 06, 09, 10, and 12), DNA was detected at some point during the follow-up period in all types of clinical samples analysed. High viral loads (Cq values ≤ 21) were observed in some saliva, rectal swab, semen, urine and faecal samples. Intermittent shedding (negative PCR results that became positive in the following time point collected) was also observed in samples such as urine or saliva (Patients 03 and 05). Importantly, MPX virus was detected in saliva from all 12 patients studied, and in some cases, at low Cq values indicative of high viral loads. In addition, the other clinical samples tested were frequently positive for MPX virus: rectal swab (11/12 cases), nasopharyngeal swab (10/12 cases), semen (7/9 cases), urine (9/12) and faeces (8/12).

**Table 3:** Clinical sample characteristics of 12 monkeypox patients in follow-up samples by collection day since symptom onset and qPCR results, Barcelona, Spain, May–June 2022 (n = 129 samples)

Patients	Days since symptom onset	Specimen type					
		Saliva n = 22	Rectal swab n = 23	Nasopharyngeal swab n = 23	Semen n = 16	Urine n = 23	Faeces n = 22
Pat. 01	12	Pos (35.7)	Pos (38.4)	Pos (34.7)	Neg	Neg	Pos (23.4)
Pat. 02	11	Pos (20.3)	Pos (17.6)	Pos (33.3)	NA	Pos (37.3)	Neg
Pat. 03	10	Neg	Pos (31.9)	Neg	Pos (36.0)	Neg	Neg
	13	Pos (35.6)	Pos (33.3)	Neg	Pos (35.7)	Neg	Neg
Pat. 04	7	Pos (35.0)	Pos (19.8)	Pos (40.0)	Pos (40.0)	Neg	Pos (24.0)
	9	Neg	Pos (22.1)	Neg	Neg	Neg	Pos (21.5)
Pat. 05	14	Neg	Pos (20.7)	Pos (37.2)	Pos (31.9)	Neg	NA
	16	Pos (37.9)	Pos (21.5)	Pos (37.3)	Pos (30.7)	Pos (27.0)	Pos (19.9)
Pat. 06	11	Pos (23.2)	Pos (25.4)	Pos (25.4)	NA	Pos (39.1)	Pos (24.7)
	12	Pos (24.7)	Pos (29.8)	Pos (25.5)	NA	Neg	Pos(26.4)
	13	Pos (25.0)	Pos (27.1)	Pos (26.4)	NA	Neg	Pos (28.3)
Pat. 07	4	Pos (24.0)	Pos (22.0)	Pos (26.9)	NA	Pos (39.3)	Pos (31.4)
	6	Pos (21.8)	Pos (19.0)	Pos (26.1)	Neg	Neg	Neg
Pat. 08	5	Pos (29.1)	Pos (30.5)	Pos (32.6)	NA	Pos (39.4)	Neg
	8	Pos (31.7)	Pos (34.4)	Pos (32.9)	NA	Neg	Neg
Pat. 09	7	Pos (29.8)	Pos (23.7)	Pos (33.5)	Pos (22.7)	Pos (24.4)	Pos (30.2)
	8	Pos (25.6)	Pos (20.7)	Pos (29.4)	Pos (20.9)	Pos (19.1)	Pos (29.8)
Pat. 10	4	Pos (31.2)	Pos (23.4)	Pos (29.0)	Pos (38.7)	Pos (40.0)	Pos (20.0)
	7	Pos (36.0)	Pos (26.0)	Pos (36.5)	Neg	Pos (40.0)	Pos (23.4)
Pat. 11	1	NA	Neg	Neg	Pos (33.0)	Pos (31.8)	Neg
	4	Pos (36.5)	Neg	Neg	Pos (38.1)	Pos (35.0)	Neg
Pat. 12	4	Pos (25.3)	Pos (20.8)	Pos (31.1)	Pos (32.1)	Pos (29.0)	Pos (20.9)
	6	Pos (25.0)	Pos (21.2)	Pos (28.8)	Pos (29.7)	Pos (26.7)	Pos (17.8)

NA: not available; Neg: monkeypox DNA not detected as no Cq value obtained; Pos: positive, monkeypox DNA detected.

Quantification cycle (Cq) values are indicated in brackets after positive results.

## Discussion

The MPX virus is a zoonotic pathogen and the most frequent orthopox virus infection in humans. Since the description of the disease in 1970 in Democratic Republic of Congo, a number of cases have been reported in endemic countries but also outside endemic areas in travellers returning from western and central Africa [2,7]. In 2003, a MPX outbreak in the United States linked to the importation of infected animals was reported [8]. The virus was then considered as an emerging infection and a potential public health threat, which unfortunately has been confirmed in subsequent MPX outbreaks [7], including the current ongoing outbreak [9]. 

In this study, we provide data on 147 clinical samples, collected at 23 time points from 12 confirmed MPX cases in Barcelona. Our knowledge on MPX virus shedding is clearly limited, but greatly needed in the current epidemiological situation, where many countries are experiencing an upsurge of cases. To our knowledge, a single report [3] has been published on this issue, reporting results on 24 samples from four cases and detected MPX DNA in semen (three patients), blood (one patient), nasopharyngeal swab (three patients), faeces (two patients) and saliva (one patient). In addition, a study from Germany [10] detected MPX virus in blood and semen of two cases. In the current outbreak, several clinical and epidemiological data support that close contact often in the context of sexual activity is driving disease transmission [11]. Thus, a detailed description of the presence of the virus in bodily fluids may shed light on the mechanisms of viral transmission.

At the time of diagnosis, MPX virus DNA was detected in swabs of skin lesions in all patients. High viral loads (Cq value range: 16–21) were observed in skin pustules. Some patients presented with additional oral, pharyngeal and rectal lesions. The ability of MPX virus infection to cause proctitis and other atypical clinical presentations warrants further research [12,13]. Interestingly, the analysis of follow-up samples showed shedding of MPX virus in a range of bodily fluids during the first 2 weeks of the illness and up to 16 days after symptom onset. 

Our results on saliva samples are of special interest. In a previous report, saliva was only tested once in a single patient [3]. Here, we find that MPX DNA was detected in saliva at some point in all 12 patients studied, in the samples collected between 4–16 days after the onset of symptoms. The other clinical samples analysed, including semen, frequently contained MPX virus DNA. We did not perform cell culture, and a clear correlation between real-time PCR and virus isolation has not been reported in existing literature. However, results from studies in animal samples that quantified MPX virus and performed cell culture indicate that virus isolation can be successful with viral loads in the range of 10^4^–10^5^ copies/ml [14]. Furthermore, during the present outbreak, MPX virus has been isolated from skin lesion samples with a Cq of 20 in one case [10]. With the low Cq values observed in our study in a variety of samples such as saliva, rectal swab, semen, urine and faecal samples, further research on the infectious potential of these bodily fluids and their potential role in disease transmission by close physical contact during sexual activity is warranted.

The initial spread of MPX in Europe seemed to be related to specific mass gathering events in Spain and Belgium [15]. Our data show that, among the 12 cases studied, history of travel to these areas was absent and only some patients reported having had close physical contact with positive MPX cases. Indeed, while the studied patient size was small, our accumulated experience with approximately 125 diagnosed cases indicates that, in some of our first cases, the history of travel to the aforementioned events was more frequent, while in subsequent cases, a history of sexual contact with someone who attended one of these events or an absence of this epidemiological linkage was more frequent (data not shown). This, together with the rising number of cases worldwide [1], supports the notion of sustained MPX transmission in the community, at least within MSM risk groups in Barcelona.

An increase in MPX cases has been linked to a decline of smallpox vaccinated population in endemic areas such as Nigeria [7]. Vaccination of high-risk household and identified close contacts is being considered as a complementary measure for the control of the current MPX outbreak by some organizations such as the UK Health Security Agency [16]. Of note, in our study, smallpox vaccination was reported in four out of the seven patients in which this information was available. Additional detailed information on the vaccine history from larger case series as well as coupled serological testing should be performed for a better understanding of the protective effect of the smallpox vaccine against the currently circulating MPX virus strain. 

## Conclusions

MPX virus transmission dynamics, similarly to other newly emerging viral infections, may need to be addressed under multidisciplinary approaches. Our results contribute to an improved understanding of a likely complex transmission puzzle and underline other immediate areas for research such as the infectivity of bodily fluids, the frequency of secondary and asymptomatic cases or the impact of social and behavioural factors affecting viral transmission. Our results may be valuable as well for diagnostic testing algorithms and public health interventions.
